# An Innovative Model for Diagnosing Lesions in Coronary Angiography Imagery Using an Improved YOLOv4 Model

**DOI:** 10.3390/bioengineering12111241

**Published:** 2025-11-12

**Authors:** Zhu Chen, Yajie Chen, Jiajia Si, Changhu Xiao, Xiaohan Liu, Chengming Wang, Fengling Chen, Yuan Guo

**Affiliations:** 1 Institute for Future Sciences & Hengyang Medical School, University of South China, Hengyang 421001, China; 2 Department of Cardiovascular Medicine, Zhuzhou Hospital Affiliated to Xiangya School of Medicine, Central South University, Zhuzhou 412007, China; 3 Hunan Key Laboratory of Biomedical Nanomaterials and Devices, Hunan University of Technology, Zhuzhou 412007, China

**Keywords:** acute myocardial infarction, coronary artery angiography, transfer learning, YOLOv4 algorithm

## Abstract

Percutaneous coronary angiography remains the diagnostic gold standard for coronary artery disease. However, the complex and high-volume nature of the imaging data renders the clinical interpretation of coronary lesions a time-consuming, labor-intensive, and inherently subjective process. This retrospective study collected and preprocessed Coronary artery angiography (CAG) image data from 408 patients with acute myocardial infarction (AMI). An improved YOLOv4 algorithm was developed, validated on standard VOC datasets, and subsequently calibrated via transfer learning on the CAG training set for automated lesion detection and classification. The model-derived lesion characteristics were then statistically correlated with the occurrence of Major Adverse Cardiovascular Events (MACEs) during patient follow-up. The improved model achieved a post-modification mean Average Precision (mAP) of 84.72% (95% CI: 83.44–85.99%) on the VOC dataset. For coronary lesion detection, the model yielded an overall mean Average Precision (mAP) of 55.01%. Importantly, lesion characteristics automatically detected by the model—specifically completely occluded lesions (Log-rank *p* = 0.003) and multibranching lesions (Log-rank *p* = 0.033)—demonstrated a significant association with the cumulative incidence of MACEs. The innovative, improved YOLOv4 model exhibits robust performance in effectively and accurately detecting and classifying coronary lesions within AMI patient angiography imagery. This study provides a valuable AI-assisted diagnostic tool and offers preliminary insights for long-term prognostic assessment by seamlessly integrating deep learning-derived anatomical features with MACEs prediction.

## 1. Introduction

Percutaneous coronary angiography is widely recognized as the gold standard for diagnosing coronary atherosclerotic heart disease, with Acute Myocardial Infarction (AMI) representing the most severe clinical manifestation of atherosclerotic heart disease [1,2]. Beyond its traditional role in diagnosis and guiding revascularization, recent evidence highlights the crucial value of coronary angiography in prognostic risk stratification for AMI patients [3,4]. Major Adverse Cardiovascular Events (MACEs)—defined as the composite outcome of subsequent myocardial infarction, heart failure, renal failure, recurrent coronary events, cerebrovascular events, and sudden death during post-AMI follow-up—have been established as the primary endpoint for assessing AMI prognosis [5,6]. A significant body of the literature confirms a strong correlation between the characteristics of coronary lesions and the long-term prognosis of AMI patients [7,8].

However, the clinical utilization of CAG images is fraught with challenges. The procedure generates a substantial volume of complex video and image data, making the subsequent interpretation of coronary lesions a time-consuming, labor-intensive, and inherently subjective undertaking that relies heavily on the operator’s experience [9]. Crucially, subtle, yet prognostically relevant, anatomical details within the videos are often challenging to perceive and may be overlooked, diminishing their potential contribution to diagnosis and risk assessment. Consequently, the development of an innovative algorithmic model for intelligent image recognition, specifically optimized for processing coronary angiography imagery, holds substantial significance for enhancing the diagnostic precision, treatment planning, and prognostic evaluation of AMI patients [10,11].

The Convolutional Neural Network (CNN) represents a foundational deep learning architecture that leverages specialized mathematical convolution operations to efficiently extract patterns and features from grid-structured data like images [12,13]. Prior studies have explored the application of CNNs for identifying and classifying pathological features in CAG images, demonstrating initial effectiveness [10,14,15]. Nonetheless, the inherent complexity of coronary angiography images, characterized by intricate vascular distribution and diverse lesion morphologies, necessitates further refinement and optimization of existing CNN-based algorithms for robust coronary lesion recognition.

Target detection algorithms constitute a critical area within CNN research, capable of simultaneously localizing and classifying objects while maintaining a commendable balance between speed and accuracy [16,17,18,19]. YOLOv4 stands out as a high-performance, single-stage target detection algorithm renowned for its efficiency and excellent compatibility with multiscale detection [20,21]. Given the multiscale nature of coronary lesions (ranging from subtle stenoses to total occlusions), we hypothesize that the YOLOv4 algorithm can be effectively adapted and applied to the video and image processing tasks inherent to coronary angiography.

**Figure 1:**
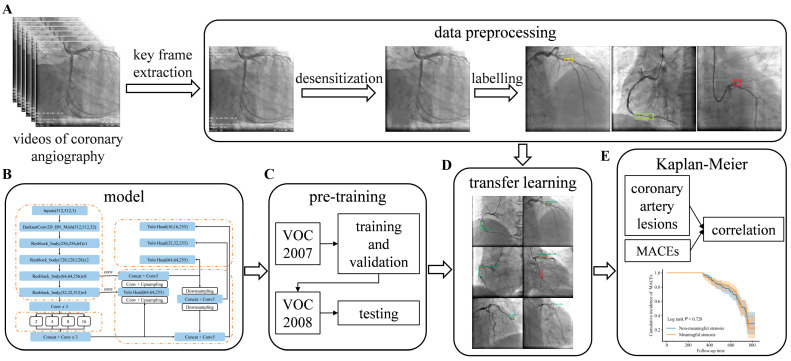
Overall workflow diagram for intelligent diagnosis and prognostic assessment based on Coronary Angiography image analysis. The study workflow encompasses five main phases, from video data processing to model construction, pre-training, transfer learning, and final prognostic analysis. (**A**) Data Preprocessing. Key frames are extracted from coronary angiography videos, undergo desensitization, and are labeled for subsequent training. Colored Labeling Key: In the “labelling” step, colored boxes are used to mark different types of coronary lesions. Dark yellow presents limited meaningful lesions; Light green presents diffuse lesions; Red presents completely occlusive lesions. (**B**) Model. The architecture utilizes the improved YOLOv4 object detection algorithm. (**C**) Pre-training. The model is trained using the VOC 2007 dataset for training and validation, and the VOC 2008 dataset for testing, to acquire robust initial weights. (**D**) Transfer Learning: The pre-trained model is fine-tuned and tested on the clinical Coronary Angiography dataset. (**E**) Kaplan-Meier Analysis. Quantitatively assesses the correlation between algorithmically derived coronary artery lesion characteristics and the incidence of MACEs for prognostic evaluation.

The primary aim of this study is twofold: (1) to establish an effective algorithmic model for the automated identification of coronary lesions in clinical angiography images; and (2) to quantitatively assess the correlation between the algorithmically derived lesion characteristics and the occurrence of MACEs post-AMI. This research intends to offer a novel, data-driven perspective for constructing sophisticated prognostic assessment models following AMI, and the overall workflow is shown in Figure 1.

## 2. Methods

### 2.1. Patient and Image Inclusion

**Figure 2:**
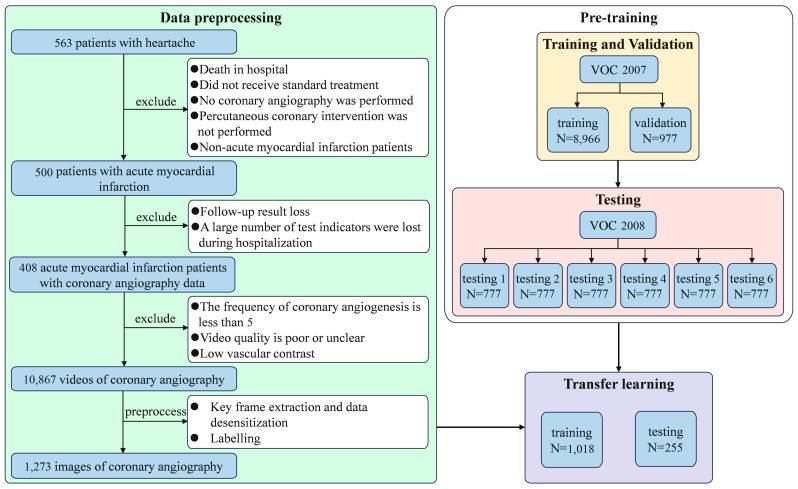
Flowchart of coronary angiography image inclusion and algorithm application.

The detailed procedure for patient inclusion and image acquisition has been extensively reported in our prior study [22]. This retrospective investigation enrolled patients diagnosed with Acute Myocardial Infarction (AMI) who underwent Coronary Angiography (CAG) at our institution between January 2017 and December 2019. Inclusion criteria were defined as, but not limited to, first-onset AMI and successful CAG procedures where primary lesion morphology could be clearly determined. Exclusion criteria comprised patients with an insufficient video count (fewer than five videos), those exhibiting poor or unclear video quality, or individuals with low contrast levels that hampered accurate lesion visibility. The final cohort for image analysis consisted of 1273 CAG images derived from 408 patients. The CAG data were originally stored in DICOM format, with each patient contributing between 5 and 51 video sequences. The resolution of each frame was standardized to 512 pixels × 512 pixels. Key frames were systematically extracted from each CAG video sequence, prioritizing frames that exhibited maximal contrast clarity, minimal motion artifact or background noise, and distinct vessel morphology to ensure optimal feature visibility for subsequent deep learning analysis. Ultimately, 1273 coronary angiography images were obtained for further analysis. The detailed process is illustrated in Figure 2.

### 2.2. Standard Data Set Application

The VOC 2007 and VOC 2008 datasets were selected for the initial pre-training and enhancement of the object detection algorithm [23]. The VOC 2007 dataset comprises 9963 images and corresponding labels, while the VOC 2008 dataset includes 4332 images and labels. Collectively, these datasets encompass 20 distinct object categories (e.g., horse, cow, car, person). Each image may contain multiple object instances and categories. The ground-truth label files are provided in XML format, detailing the object categories and their respective bounding box coordinates within the image files.

### 2.3. Desensitization and Annotation of Coronary Angiography Dataset

Following the acquisition of the patient’s coronary angiogram images, desensitization was performed using MicroDicom (MicroDicom DICOM viewer 2023.3) [24]. This process involved the removal of the patient’s name, gender, age, and hospitalization information from the coronary angiogram video, which was to protect the patient’s privacy and enhance the accuracy of the test.

Next, coronary lesions were defined as meaningful stenosis (coronary artery stenosis of more than 50% of the vessel diameter), diffuse stenosis (meaningful stenosis exceeding 20 mm in length at the same location), and complete occlusion (complete occlusion of the coronary artery diameter), respectively. Coronary lesions in coronary angiography were manually labeled using the LabelImg program package to obtain the necessary data and labels required for the target detection model. The labels for the target detection algorithm adopted a bounding box format, encompassing information about the lesion location and category. And the label files were saved in XML format.

To ensure labeling accuracy, the process involved two steps. In the initial step, two physicians simultaneously labeled the coronary angiography images. Upon completion, the degree of overlap of the bounding boxes labeled by the two physicians was assessed using Intersection of Union (IOU). If the IOU value exceeded 0.8 and the coronary lesion categories were consistent, the bounding box would be expanded to a rectangular shape, preserving the coronary lesion category after the overlap of the two boxes. In cases of inconsistent labeling, a second round of labeling was initiated. In the second step, an authoritative expert reviewed the results of the initial labeling round and re-labeled the coronary lesions.

### 2.4. Model Construction

The methodology for automated Computer-Aided Diagnosis (CAD) was executed through a three-phase workflow: enhancing the YOLOv4 algorithm for effective recognition of small- and medium-sized lesions, followed by pre-training and validation using the VOC datasets, and concluding with transfer learning and fine-tuning on the coronary angiography (CAG) dataset.

**Figure 3:**
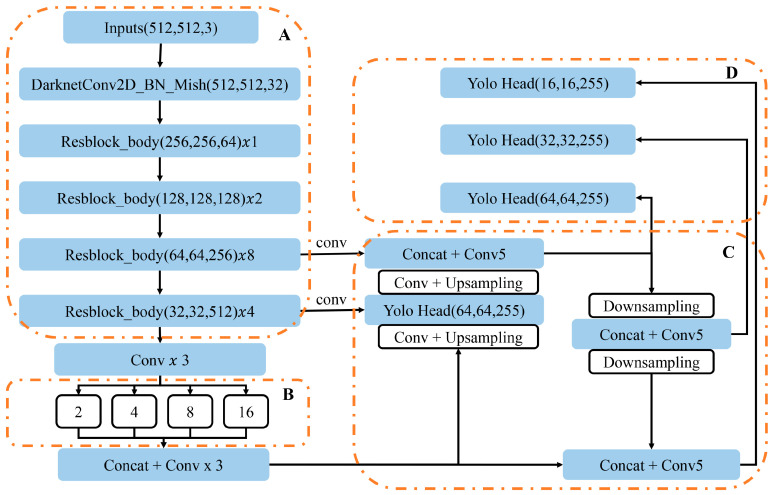
Diagram of the improved YOLOv4 model. (**A**) CSPDarknet53 Backbone. The feature extraction network that processes the Inputs (512, 512, 3) image and yields features at various scales. (**B**) Modified Spatial Pyramid Pooling (SPP) Module. The pooling layer sizes are optimized to 1, 2, 4, 8, and 16, enhancing the model’s multiscale feature aggregation capacity for accurate localization of variably sized lesions. (**C**) Path Aggregation Network (PANet). The feature aggregation modules that fuse high-level and low-level features through convolution, upsampling, downsampling, and concatenation to enrich the feature map. (**D**) YOLO Detection Heads. The standard detection heads that output final predictions (bounding boxes and classification) at three different scales (e.g., (16, 16, 255), (32, 32, 255), and (64, 64, 255)).

In the initial phase, we developed an improved YOLOv4 target detection algorithm. This architecture utilizes the CSPDarknet53 backbone, augmented feature extraction modules including Spatial Pyramid Pooling (SPP) and Path Aggregation Network (PANet) (as shown in Figure 3A), and the standard YOLO head (Figure 3B). To optimize the model for CAG images, we increased the input size to 512 × 512 pixels, allowing the CSPDarknet53 backbone to yield 1024 features at a 16 × 16 pixel scale (Figure 3C). A critical modification was the optimization of the SPP module’s pooling layer sizes to 1, 2, 4, 8, and 16 (Figure 3D), specifically designed to boost multiscale feature aggregation capacity for accurate localization of variably sized coronary lesions.

In the second stage, the enhanced YOLOv4 algorithm was pre-trained and validated using the VOC 2007 and VOC 2008 datasets to acquire robust initial model weights. The VOC 2007 dataset (9963 images) was split 9:1 for training and validation, while the VOC 2008 dataset (4332 images) was partitioned into six equal subsets for testing (Figure 2). Pre-training was conducted for 100 epochs (batch size 4), employing an initial learning rate of 0.01 (minimum 0.0001) updated via a cosine decay schedule, along with an early stopping mechanism (training terminated if loss did not improve after 10 non-improving epochs on the validation set). The completed pre-trained model was subsequently tested on the six VOC subsets, and the predicted Average Precision for each object category, as well as the overall mean Average Precision, was computed.

In the final stage, the pre-trained model underwent rigorous re-training and testing on the clinical coronary angiography (CAG) dataset via a two-phase transfer learning protocol. The workflow commenced by splitting the labeled CAG dataset into a training set and a testing set at an 8:2 ratio (Figure 2). The initial round of training involved importing the pre-trained model weights while the backbone feature extraction network (CSPDarknet53) was frozen. This phase was executed for 100 epochs with a batch size of 4. The optimization used an initial learning rate of 0.01 with a minimum value of 0.0001, employing a cosine decay schedule to dynamically update the rate; once the rate dropped below the minimum, further decay was discontinued. The second round of training began by unfreezing the backbone feature extraction network to allow for full fine-tuning of the entire model. An additional 100 epochs of training were performed using the weights optimized from the first round. The batch size was reduced to 2, the initial learning rate was set to 1 × 10^−4^, and the minimum value was set to 1 × 10^−6^. Similar to the initial round, the learning rate was updated using the cosine decay schedule under these revised parameters. Finally, the fully fine-tuned model was subjected to a rigorous evaluation using the held-out CAG test set. The assessment focused on observing the Average Precision values of the model for the detection of each specific lesion type and the overall mean AP (mAP) for all lesion categories.

### 2.5. Indicators for Model Evaluation

The performance of the object detection algorithm was primarily assessed using standard metrics, including Accuracy, Precision, Recall, and Average Precision (AP), with AP being recognized as the optimal single metric for object detection tasks [25]. As shown in Table 1, TP represents true positives, indicating cases where the predicted and true results are both positive. TN represents true negatives, indicating cases where the predicted and true results are both negative. FN represents false negatives, indicating cases where the predicted result is negative, but the true result is positive. FP represents false positives, indicating cases where the predicted result is positive, but the true result is negative. The formulas for calculating accuracy, precision, and recall are given by Equations (1), (2), and (3), respectively.


(1) $$\mathrm{Accuracy}=\frac{TP+TN}{TP+FN+TN+FP}$$

(2) $$\mathrm{Precision}=\frac{TP}{TP+FP}$$

(3) $$\mathrm{Recall}=\frac{TP}{TP+FN}$$

The AP is defined as the area under the precision-recall curve. The formula for calculating AP is given by Equation (4). In object detection problems, each object category is associated with an AP value

(4) $$AP=\int_{0}^{1}f(x)d(x)$$

where f(x) represents the mapping from recall to precision. Mean Average Precision (mAP) represents the weighted average of the AP values across all object categories.

### 2.6. Assessment of MACEs Based on Coronary Lesions

To assess the clinical significance of the automatically detected lesions, patients were dichotomized into two subgroups based on the presence or absence of specific coronary artery lesion characteristics: meaningful stenosis, diffuse stenosis, complete occlusion, or multibranch lesions. Kaplan–Meier survival analysis was subsequently employed to statistically compare the differences in Major Adverse Cardiovascular Event (MACE) occurrence between the corresponding subgroups for each lesion type. A Log-rank *p* < 0.05 was established as the threshold for statistical significance.

## 3. Results

### 3.1. Preprocessing Results of Coronary Angiography Images

**Figure 4:**
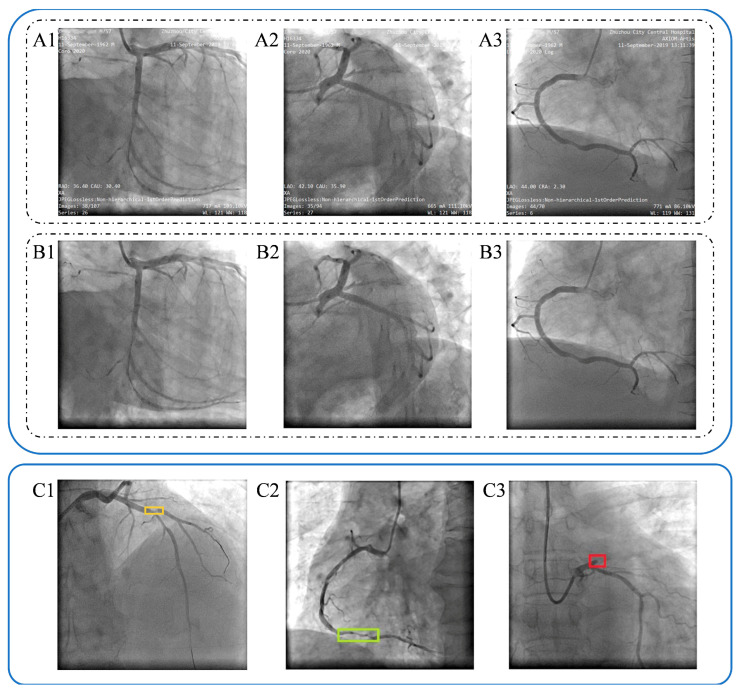
Coronary angiography image desensitization and labelling. (**A1**–**A3**). Original images of coronary angiography from patients. (**B1**–**B3**). Desensitization of relative original images. (**C1**–**C3**). Labelling of the narrowed coronary arteries image, dark yellow in (**C1**) presents limited meaningful lesion, light green in (**C2**) presents diffuse lesion, red in (**C3**) presents completely occlusive lesions.

A total of 563 patients diagnosed with Acute Myocardial Infarction (AMI) were initially screened. Following the application of predefined exclusion criteria and subsequent accounting for patient loss to follow-up, a final cohort of 408 patients was included for comprehensive analysis. From this final cohort, 1273 distinct coronary angiography (CAG) images were compiled and integrated into the image analysis study. The detailed clinical characteristics and statistical outcomes regarding the follow-up incidence of Major Adverse Cardiovascular Events (MACEs) for this patient population have been previously reported [22]. To prepare the data for the deep learning model, all acquired CAG images underwent desensitization (removal of patient-identifying information) prior to lesion annotation. Coronary lesions were manually labeled, with a definition of significant stenosis established as a narrowing of the coronary artery diameter exceeding fifty percent (≥50). The final annotated images, illustrating the various lesion types, are presented in Figure 4.

### 3.2. YOLOv4 Model Modification and Evaluation

**Figure 5:**
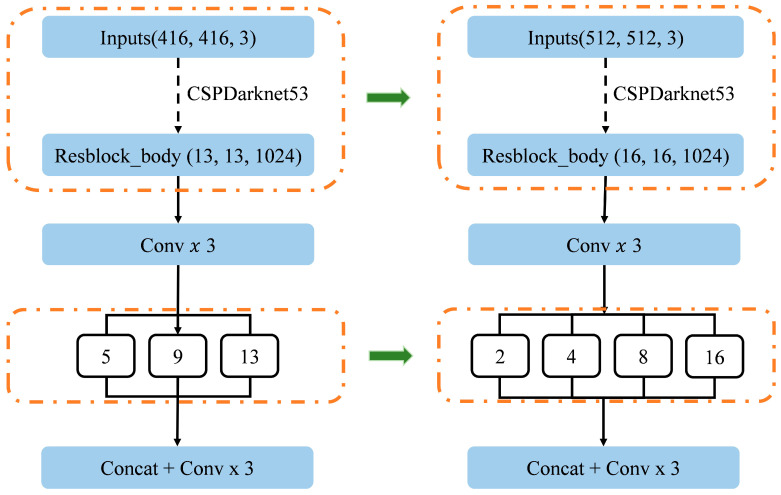
Schematic diagram of SPP module before and after improvement.

To achieve the requisite high detection accuracy for processing complex coronary angiography (CAG) images, we implemented targeted structural modifications to the baseline YOLOv4 architecture. These enhancements were specifically engineered to address the challenges posed by multiscale lesions. The main modifications were twofold: first, the model’s input resolution was upscaled from 416 × 416 pixels to 512 × 512 pixels, a change that provides a richer contextual and spatial feature map crucial for resolving small-sized lesions. Second, we significantly optimized the Spatial Pyramid Pooling (SPP) module by replacing the standard pooling layer sizes with a refined sequence of kernel dimensions: 1 × 1, 2 × 2, 4 × 4, 8 × 8, and 16 × 16. This particular adjustment was designed to fundamentally boost the network’s multiscale feature representation capability, thereby enhancing its robustness and accuracy in detecting coronary lesions that exhibit wide variations in size. The schematic diagrams of the SPP module before and after improvement are shown in Figure 5.

**Figure 6:**
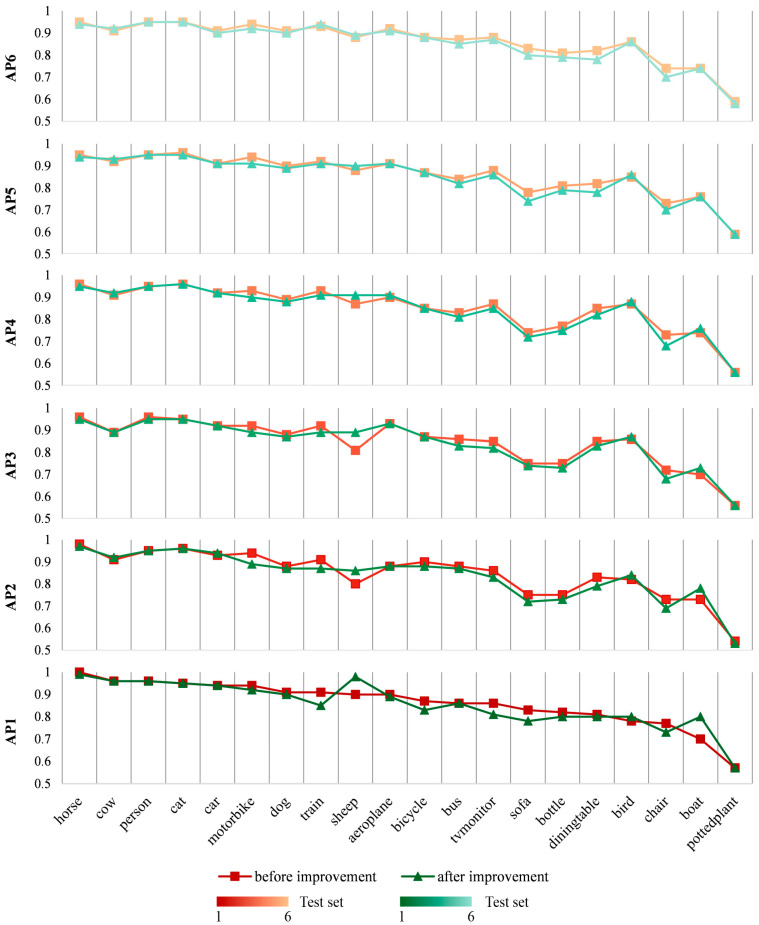
Line graph showing validation of improved YOLOv4 model on the VOC 2008 dataset. The VOC 2008 dataset was randomly divided into six equal parts for testing the pre-trained model. The improved model’s training batch was set to 100, with a batch size of 4. The initial learning rate was set to 0.01, with a minimum value of 0.0001. Average precision (AP) values for each object category were computed.

To validate the efficacy of the structural optimizations, the modified model’s performance was first assessed on the established VOC datasets. The mean Average Precision (AP) for the pre-modification baseline model was 85.43% (95% CI: 84.12–86.73%). Following the implementation of the structural adjustments, the AP was measured at 84.72% (95% CI: 83.44–85.99%). This comparison demonstrated that the improved architecture maintained a substantially comparable detection performance for the large-scale object categories characteristic of the VOC dataset, showing no significant increase in performance. This outcome is consistent with the primary design objective: the architectural modifications were specifically engineered to enhance the model’s ability to recognize small-scale objects (i.e., coronary lesions) rather than providing a generalized improvement for large-object detection tasks. The detailed verification results are shown in Figure 6.

### 3.3. Identification of Coronary Lesions by the Improved YOLOv4 Model

Following the successful pre-training of the optimized YOLOv4 algorithm on the VOC dataset, we performed subsequent specialized training and fine-tuning on the dedicated coronary angiography (CAG) image set via transfer learning. The comprehensive testing results clearly demonstrated the model’s high capability to classify and localize distinct coronary lesions: the improved YOLOv4 model achieved Average Precision (AP) values of 56.41%, 58.28%, and 50.35% for the detection of meaningful stenosis, diffuse stenosis, and complete occlusive lesions, respectively. Critically, the overall mean Average Precision (mAP) for detecting all three categorized coronary artery lesions reached 55.01%.

**Figure 7:**
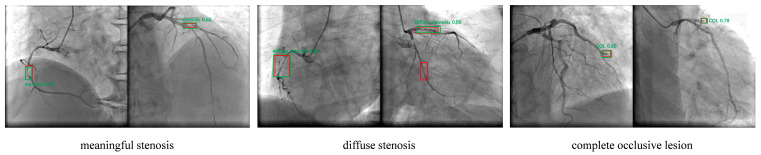
Comparison of the improved YOLOv4 model for the identification of different lesions in coronary angiography images. The red box represents the true value of manual labeling; the green box represents the predicted value of the lesion by the improved YOLOv4 model.

For a qualitative assessment and visual confirmation of the model’s performance in localizing and classifying the pathology, representative prediction results for significant stenosis, diffuse stenosis, and complete occlusive lesions are illustrated in Figure 7. In these visualizations, the red bounding boxes denote the manually annotated ground truth, while the green bounding boxes represent the model-predicted values. Furthermore, the model exhibited high confidence in its predictions, as demonstrated by the confidence scores across various lesion types and locations: for significant stenosis, the detection confidence levels in the right coronary artery and left anterior descending artery were 0.79 and 0.82, respectively; detection of diffuse stenosis yielded confidence scores of 0.81 and 0.80; and for complete occlusive lesions, the scores were 0.80 and 0.78. These quantitative metrics and qualitative visualizations collectively affirm that our proposed transfer learning methodology successfully adapted the improved YOLOv4 model to the specialized domain of coronary angiography images, thereby enabling the accurate, robust, and effective identification of various types of coronary vascular pathology.

### 3.4. Analysis of the Correlation Between the Incidence of MACEs and the Prediction of Different Types of Lesions in Patients with AMI

To further elucidate the clinical significance of coronary lesions and their potential for prognostic assessment in AMI, we conducted a survival analysis correlating different lesion types with the incidence of Major Adverse Cardiovascular Events (MACEs).

**Figure 8:**
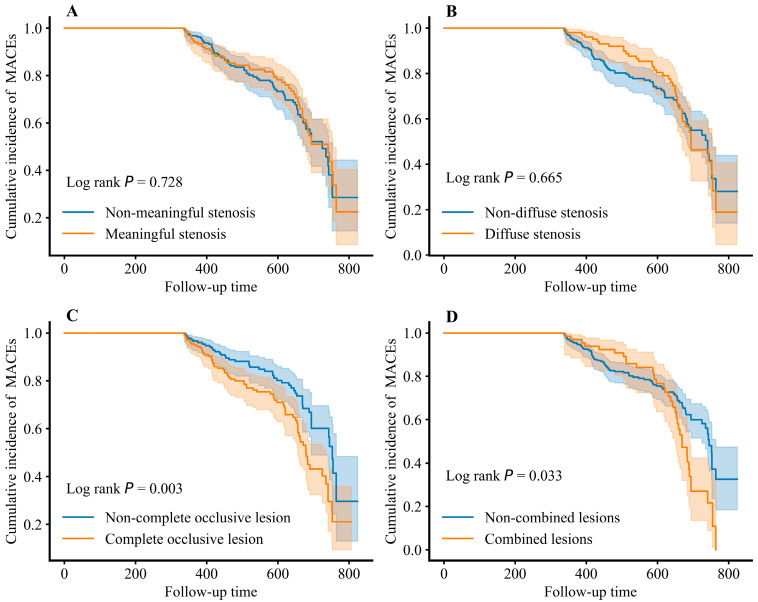
Association between coronary lesions and MACE occurrence in AMI patients. (**A**) Association between meaningful stenosis lesions and MACEs. (**B**) Association between diffuse stenosis lesions and MACEs. (**C**) Association between complete occlusive lesions and MACEs. (**D**) Association between combined lesions and MACEs. AMI: acute myocardial infarction, MACEs: major adverse cardiovascular events. The shaded area around the curve represents the 95% confidence interval.

As detailed in the Kaplan–Meier survival curves shown in Figure 8, the Log-rank *p* values for predicting MACEs based on lesion categories were: 0.728 for meaningful stenosis, 0.665 for diffuse stenosis, and 0.003 for chronic total occlusion (CTO). These results indicate a statistically significant association only for chronic total occlusion.

Furthermore, we performed an analysis for patients presenting with multivessel disease (combined lesions), defined as the presence of the aforementioned three lesion types in more than one coronary vessel. The survival analysis comparing patients with single-vessel disease versus multivessel disease yielded a Log-rank *p* value of 0.033 regarding MACE occurrence.

These findings collectively suggest that, among individual lesion types, complete occlusive lesions (CTOs) exhibit the most robust predictive value for MACEs in AMI patients. Moreover, multivessel lesions demonstrate a superior prognostic capability for MACEs incidence when compared to pathology confined to a single vessel.

## 4. Discussion

The primary objective of this investigation was to develop and rigorously validate an innovative deep learning model for the automated detection and classification of coronary lesions in angiography imagery, and subsequently to explore the clinical utility of these detected features for long-term prognostic assessment. The core finding of this study is the successful development and validation of an optimized YOLOv4 model, which achieved an acceptable overall mean Average Precision (mAP) of 55.01% on the challenging coronary angiography (CAG) dataset. We acknowledge that the model’s performance on the VOC dataset showed no significant improvement for large-scale objects compared to the baseline, which is attributed to the intentional architectural modifications (optimized SPP module and increased input size) specifically engineered to enhance small-scale object detection relevant to coronary lesions. Crucially, the lesion characteristics automatically extracted by the model—particularly complete occlusive lesions (CTOs)—were found to be significantly associated with the long-term incidence of Major Adverse Cardiovascular Events (MACEs) in AMI patients. Furthermore, multivessel lesions demonstrated a superior prognostic capability for MACEs incidence when compared to pathology confined to a single vessel. These results provide compelling support for the potential of advanced object detection algorithms to serve as a powerful tool for AI-assisted diagnosis and prognostic risk stratification in clinical cardiology.

Coronary angiography remains one of the most crucial methods for diagnosing and assessing the prognosis of AMI patients [3,8,26]. However, current reliance on manual interpretation leads to low efficiency and high subjectivity, especially when identifying small or dynamic lesions. While prior deep learning studies have utilized Convolutional Neural Networks (CNNs) for vessel segmentation [27] or focused on higher-quality Coronary CT Angiography (CCTA) data [28,29], our study uniquely focuses on the more complex and challenging CAG video data via the YOLOv4 target detection algorithm, recognized for its superior efficiency in real-time detection. In contrast to conventional CNN algorithms, our approach selected YOLOv4, which we enhanced to address the common deficiency in detecting small-scale information in complex medical images. We optimized the YOLOv4 algorithm’s CSPDarknet53 and SPP modules, aligning them with the characteristics of CAG images. While other studies have also improved YOLOv4 using SPP to enhance feature extraction for small objects, such as masks [30], the superior clarity and signal-to-noise ratio of those general images make detection easier than in CAG. Furthermore, our modifications contrast with approaches like Scaled-YOLOv4-HarDNet [31], as our enhancement maintains excellent detection performance for large-scale objects while also demonstrating good performance in identifying small lesions. Our study also pioneered the combined approach of transfer learning with the enhanced YOLOv4 algorithm to optimize recognition in this specific medical domain, demonstrating that pre-trained weights significantly aid generalization to unseen, complex clinical data [32].

From a translational and health economics perspective, the AI-assisted diagnostic system offers considerable benefits. Artificial intelligence, digital medicine, and models based on well-developed databases significantly simplify work and are undoubtedly the future of medicine [33]. By providing rapid and automatic identification of complex lesions, the system can substantially reduce the reliance on time-consuming manual assessment, thereby minimizing the inherent inter-observer subjectivity. This automation holds the potential to reduce the necessity for subsequent cost-intensive imaging modalities [34]. And by streamlining the initial diagnostic pathway, may also help minimize patient and operator radiation exposure. These demonstrated cost-effectiveness benefits are pivotal factors in the evaluation and adoption of new diagnostic strategies for coronary artery disease.

Despite these advances, this study is subject to several methodological limitations. Firstly, the research is constrained by its single-center, retrospective design and the relatively modest size of the CAG dataset, factors that inherently limit the generalizability and external validity of our findings. Secondly, the current analysis lacks a direct comparative evaluation against other state-of-the-art deep learning models, which somewhat curtails the robustness of the performance claims. Future work will focus on comprehensively addressing these limitations. Performing rigorous external validation in a large, multi-center, and diverse patient cohort to substantiate and enhance the model’s ultimate clinical applicability.

## 5. Conclusions

Acute myocardial infarction (AMI) poses a significant threat to global health, and despite continuous advancements in clinical practice, there remains a critical need to enhance patient prognosis. Recent research highlights the indispensable value of coronary angiography images in the management and prognostication of AMI. Recognizing the inherent limitations of conventional angiographic analysis and current image processing techniques, our study was necessitated by the requirement for a novel, intelligent model capable of precise coronary lesion identification.

In this pioneering work, we successfully developed and validated an innovative deep learning approach. We employed transfer learning in conjunction with an enhanced YOLOv4 object detection algorithm to effectively and accurately detect and classify coronary lesions directly from the angiography images of AMI patients. The modified YOLOv4 algorithm demonstrated superior proficiency in lesion recognition, exhibiting particular efficiency in identifying diffuse stenotic lesions.

Critically, we leveraged this model to assess the correlation between automatically detected lesion types and post-AMI outcomes. Our findings reveal a strong and significant association between the presence of total occlusive lesions and combined lesions in AMI patients and the subsequent occurrence of Major Adverse Cardiovascular Events (MACEs).

This research not only introduces a vital and efficient AI-assisted diagnostic tool for coronary lesion identification but also establishes a preliminary algorithmic foundation and crucial research evidence for seamlessly integrating deep learning-derived image features with long-term clinical prognosis prediction. This framework paves the way for constructing a robust, automated AMI patient prognostic model based on objective angiographic lesion characteristics.

## Figures and Tables

**Table 1:** Confusion matrix.

	Predict Positive Categories	Predict Negative Categories
True positive label	TP	FN
True negative label	FP	TN

## Data Availability

The original contributions presented in this study are included in the article. Further inquiries can be directed to the corresponding author.
